# Synthesis and in vitro characterization of the genotoxic, mutagenic and cell-transforming potential of nitrosylated heme

**DOI:** 10.1007/s00204-020-02846-8

**Published:** 2020-07-15

**Authors:** Tina Kostka, Jörg Fohrer, Claudia Guigas, Karlis Briviba, Nina Seiwert, Jörg Fahrer, Pablo Steinberg, Michael T. Empl

**Affiliations:** 1 Institute for Food Toxicology, University of Veterinary Medicine Hannover, Hannover, Germany; 2 Present Address: Institute of Food Science and Human Nutrition, Leibniz University Hannover, Hannover, Germany; 3 Institute of Organic Chemistry, Leibniz University Hannover, Hannover, Germany; 4 Max Rubner-Institut, Federal Research Institute of Nutrition and Food, Karlsruhe, Germany; 5 Division of Food Chemistry and Toxicology, Department of Chemistry, Technical University of Kaiserslautern, Kaiserslautern, Germany

**Keywords:** Nitrosylated heme, Processed red meat, Colon cancer, Genotoxicity, Mutagenicity

## Abstract

Data from epidemiological studies suggest that consumption of red and processed meat is a factor contributing to colorectal carcinogenesis. Red meat contains high amounts of heme, which in turn can be converted to its nitrosylated form, NO-heme, when adding nitrite-containing curing salt to meat. NO-heme might contribute to colorectal cancer formation by causing gene mutations and could thereby be responsible for the association of (processed) red meat consumption with intestinal cancer. Up to now, neither in vitro nor in vivo studies characterizing the mutagenic and cell transforming potential of NO-heme have been published due to the fact that the pure compound is not readily available. Therefore, in the present study, an already existing synthesis protocol was modified to yield, for the first time, purified NO-heme. Thereafter, newly synthesized NO-heme was chemically characterized and used in various in vitro approaches at dietary concentrations to determine whether it can lead to DNA damage and malignant cell transformation. While NO-heme led to a significant dose-dependent increase in the number of DNA strand breaks in the comet assay and was mutagenic in the HPRT assay, this compound tested negative in the Ames test and failed to induce malignant cell transformation in the BALB/c 3T3 cell transformation assay. Interestingly, the non-nitrosylated heme control showed similar effects, but was additionally able to induce malignant transformation in BALB/c 3T3 murine fibroblasts. Taken together, these results suggest that it is the heme molecule rather than the NO moiety which is involved in driving red meat-associated carcinogenesis.

**Electronic supplementary material:**

The online version of this article (10.1007/s00204-020-02846-8) contains supplementary material, which is available to authorized users.

## Introduction

Several epidemiological studies suggest that a direct correlation between the incidence of colorectal cancer (CRC) and the consumption of red and processed red meat, but not white meat, exists in industrialized nations (English et al. 2004; Larsson et al. 2005; Norat et al. 2005; WCRF/AICR 2007; Vargas and Thompson 2012; Bray et al. 2018). For instance, Orlich et al. (2015) compared CRC incidence in individuals consuming a non-vegetarian or a pescovegetarian diet and concluded that the consumption of a pescovegetarian diet significantly reduces cancer risk and that meat may be one of the most important factors in nutrition-associated CRC. Depending on the method of preparation, all types of meat—i.e. red as well as white meat and fish—may contain potentially carcinogenic compounds such as polycyclic aromatic hydrocarbons (PAHs; Vanmaanen et al. 1994; Kazerouni et al. 2001; Sinha et al. 2005; Demeyer et al. 2016) or heterocyclic aromatic amines (HCAs; Hasegawa et al. 1993; Shirai et al. 1995; Ni et al. 2008; Puangsombat et al. 2012). However, such heat-induced contaminants are rather unlikely to play a prominent role in increasing CRC risk specifically associated with red/processed red meat, as (1) they have been found to occur in all types of heated meat, i.e. also in white meat, which is not associated with CRC formation, and (2) their chemical structure often precludes that significant amounts are taken up by colonic cells. For instance, in the case of HCAs, bioavailability has been shown to be typically around 1–2% (Watkins et al. 1991; Nicken et al. 2010). In addition, extremely high doses are needed to induce carcinogenic effects in vivo (reviewed by Stavric 1994) and no malignant cell transformation has been shown to occur in vitro (Nicken et al. 2015). In the case of the PAH lead compound benzo[*a*]pyrene (B*a*P), a case–control study performed by Helmus et al. (2013) showed that the control group was exposed to higher B*a*P concentrations derived from white meat than the group comprising the actual CRC cases. Moreover, results from another epidemiological study have failed to demonstrate a consistent link between PAH ingestion and CRC (Tabatabaei et al. 2010). On the contrary, it has been suggested that mutagenic factors directly arising from red meat itself may in fact play a role in CRC development (Helmus et al. 2013).

In line with this hypothesis, the International Agency for Research on Cancer (IARC) has recently classified the consumption of red meat as probably carcinogenic to humans (Group 2A) and the consumption of processed meat as carcinogenic to humans (Group 1; Bouvard et al. 2015). Up to now, the consumption of white meat has not been evaluated by IARC regarding its carcinogenicity and meta-analyses suggest that its consumption does not increase CRC risk (Huxley et al. 2009; Carr et al. 2016; Etemadi et al. 2018). In fact, the above-mentioned evidence indicates that red meat may contain endogenous genotoxic constituents and several hypotheses have been brought forward to explain the discrepancy between red and white meat regarding their antithetical association with CRC, with one being the significantly lower heme content in white meat when compared to red meat (Lombardi-Boccia et al. 2002; Vanden Bussche et al. 2014; Pretorius et al. 2016).

Heme is a rather large (616 Da) porphyrin-iron complex (Wright and Nair 2012), whose main function consists in transporting oxygen through the body as a component of hemoglobin in red blood cells (Carpenter and Mahoney 1992; Alayash et al. 2001). In spite of its important physiological function, heme may promote the formation of CRC by different mechanisms, including the induction of hyperproliferation and/or the increased formation of genotoxic compounds (reviewed by Seiwert et al. 2020). A diet rich in heme (i.e. abundant in red meat) has been shown to increase the occurrence of reactive lipoperoxides (Bastide et al. 2015) and to promote the growth of azoxymethane-induced preneoplastic aberrant crypt foci in the colon of rats (Pierre et al. 2003, 2004). Further evidence for a carcinogenic potential emanating from heme was contributed by Gilsing et al. (2013), who reported a heme-associated increase in the incidence of *APC* and *KRAS* mutations in a cohort study performed in The Netherlands. The higher heme content in red meat might also be responsible for the presumed carcinogenicity of processed red meat. The term “processed” or “cured” commonly refers to the addition of nitrite or nitrate salts to (red) meat, which directly or endogenously react with various meat components (e.g. heme) to form *N*-nitroso compounds (NOCs) such as nitrosylated heme (NO-heme), which is also known as the “cooked cured meat pigment” (Stevanovic et al. 2000; Kuhnle et al. 2007; Honikel 2008; Sun et al. 2009). The bonding of nitric oxide to the central iron atom of the heme molecule is a pH-dependent process (Hornsey 1956). For this reason, it is assumed that the slightly acidic conditions of prevailing in the proximal colon (pH 5.4–5.9; Payne et al. 2012; Moon et al. 2016) may trigger the release of nitric oxide, which, in addition to the above-mentioned genotoxic properties intrinsic to the heme moiety, could induce further pro-carcinogenic effects (Cupid et al. 2004; Gottschalg et al. 2007). This hypothesis is supported by results coming from a human nutrition study that showed higher fecal NOC concentrations and an increase in the number of DNA adducts in exfoliated colonic cells after red meat consumption, whereby both effects were positively correlated (Lewin et al. 2006). This suggests that NO-heme may be a very important factor in the context of processed red meat-related CRC development.

Although NO-heme has been detected in the gut of human volunteers that consumed a meat-rich diet (Kuhnle et al. 2007), its potential mutagenic effects as well as its involvement in (colorectal) carcinogenesis are still unclear, as demonstrated by the lack of scientific studies exploring the potential association of (pure) NO-heme with CRC. This might be explained by the fact that studies on specific biological/toxic effects of NO-heme are difficult to perform due to its insolubility in aqueous media as well as in typical cell culture-compatible solvents (e.g. ethanol or dimethyl sulfoxide [DMSO]) and its reduced stability when exposed to light and atmospheric oxygen levels (Andersen and Skibsted 1992; Jankiewicz et al. 1994; Moller et al. 2000). Moreover, NO-heme is not commercially available and most NO-heme-containing solutions synthesized according to various elder protocols (Killday et al. 1988; Jankiewicz et al. 1994; Pegg and Shahidi 1996; Soltanizadeh and Kadivar 2012) do not only contain the pure compound, but also a mixture of potentially toxic and/or mutagenic substances as well as reaction by-products (e.g. nitrite; Stevanovic et al. 2000).

Therefore, the aim of the present study was to generate purified NO-heme and to evaluate its potential DNA-damaging and cell-transforming effects using various in vitro approaches, in order to shed more light on its hypothetical role in red meat-associated carcinogenesis.

## Materials and methods

### Cell culture

BALB/c 3T3 clone A31-1-1 cells from the laboratory of M. Umeda (Hatano Research Institute, Japan) were kindly provided by Dr. A. Poth (Knoell Germany GmbH, Mannheim, Germany). The cells were cultured in minimum essential medium (MEM) supplemented with 10% (v/v) fetal calf serum (FCS), 2 mM (v/v) l-glutamine, 100 µg ml^−1^ streptomycin and 100 IU ml^−1^ penicillin (all components were obtained from Biochrom [Merck], Berlin, Germany). Chinese hamster ovary cells (subclone K1, CHO-K1-BH4) were kindly provided by B. J. Phillips in the context of the International Food Irradiation Project (IFIP; Ministry of Agriculture, Fishery and Food, UK 1984) and cultured in McCoy’s 5A medium supplemented with 10% (v/v) FCS, 100 IU ml^−1^ penicillin and 100 µg ml^−1^ streptomycin (all components were obtained from Life Technologies, Darmstadt, Germany). The human colon adenocarcinoma cell line Caco-2 was purchased from the German Collection of Microorganisms and Cell Cultures (DSMZ; Braunschweig, Germany). These cells were cultured in MEM Eagle’s (EMEM) medium supplemented with 1% (v/v) non-essential amino acids, 2 mM (v/v) l-glutamine, 50 IU ml^−1^ penicillin G, 50 µg ml^−1^ streptomycin (all components were obtained from Lonza, Verviers, Belgium) and 10% (v/v) FCS obtained from Biowest (Nuaille, France). All cell lines were kept at 37 °C in a humidified atmosphere containing 5% CO_2_.

### Chemicals and control solutions

Sodium nitrite (Carl Roth, Karlsruhe, Germany) and dehydroascorbic acid (DHAA; Sigma-Aldrich, Schnelldorf, Germany) were dissolved and diluted in ultrapure water. The nitrite concentration used in the various in vitro assays is based on the limit of quantification (LOQ) of the method used to quantify the residual nitrite content in the purified NO-heme solution prepared in the present study (see below). The concentration of DHAA applied in the various test systems was estimated by dividing the starting concentration of ascorbic acid in the NO-heme solution as published by Soltanizadeh and Kadivar (2012) (123.08 mM; see below) by the purification-based dilution factor calculated for nitrite under the assumption that DHAA is fully oxidized during the nitrosylation reaction. In order to obtain adequate solvent controls for NO-heme and hematin, acetone (99.7%; Carl Roth) was diluted in ultrapure water (acetone/H_2_O) or in 20 mM NaOH (acetone/NaOH) (Carl Roth) to a final concentration of 80% (v/v) in the case of the NO-heme solution and the hematin solution, respectively. Further chemicals used are mentioned in the respective assay sections below.

### Preparation and purification of heme solutions

The chemical structure of the different heme species mentioned in this study is shown in Supplementary Fig. 1a. Impure NO-heme was prepared as previously described by Soltanizadeh and Kadivar (2012). Briefly, 6.52 mg porcine hemin (Sigma-Aldrich) were dissolved in 1.88 ml 0.1 M NaOH and diluted in 8 ml acetone. Then, in order to obtain a 1 mM hemin solution, 120 µl of a 12.02 M HCl solution (Carl Roth) were added. Finally, heme nitrosylation was achieved by the stepwise addition of 217 mg ascorbic acid (Carl Roth; final concentration: 123.08 mM) and 138 mg sodium nitrite (final concentration: 200 mM) to the prepared hemin solution.

**Fig. 1:**
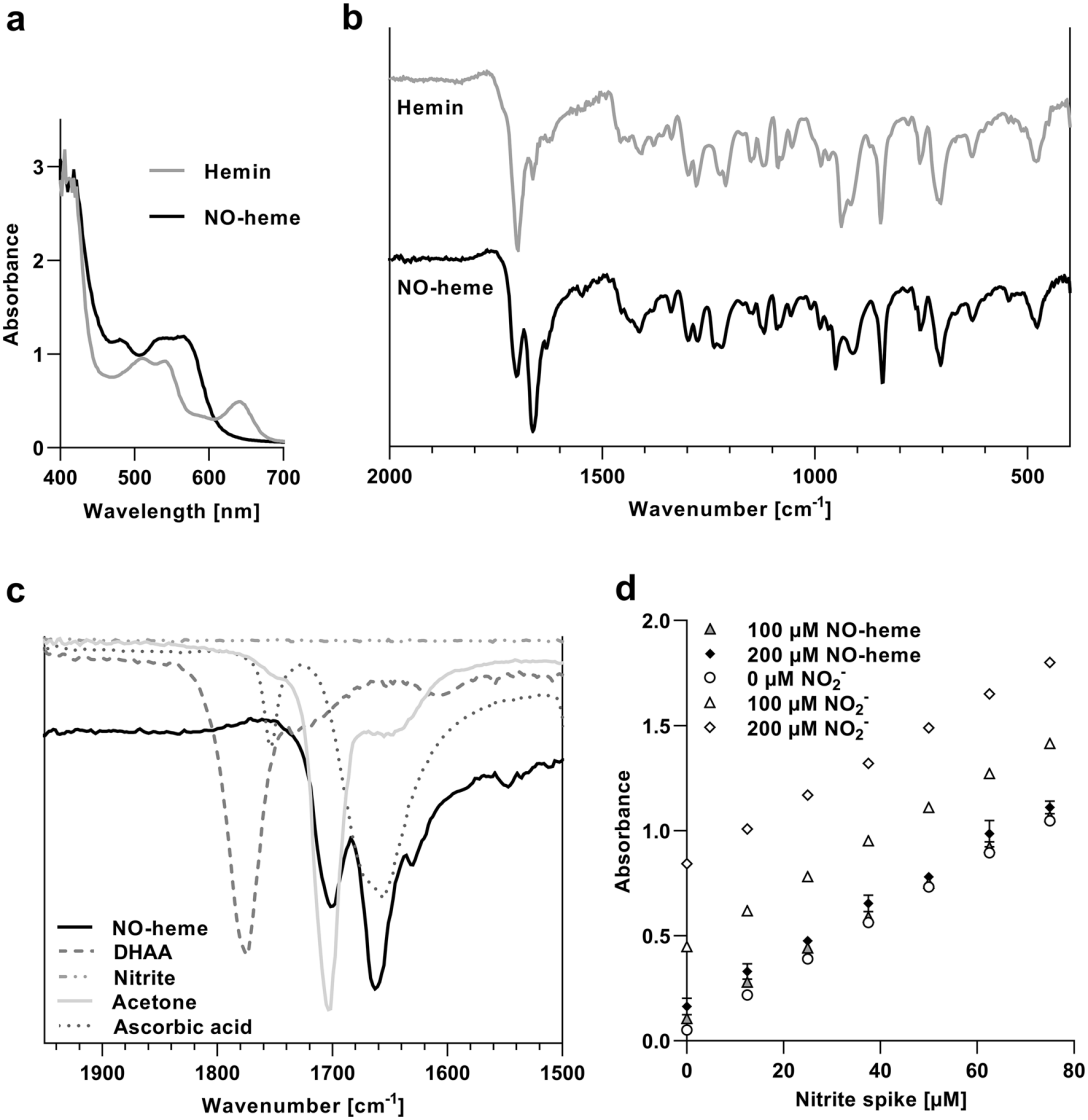
Chemical characterization of NO-heme. **a** UV–Vis and **b** IR spectra of different heme species. **c** IR spectrum of NO-heme compared to spectra of educts, by-products and solvents used in the nitrosylation reaction. **d** Detection of nitrite in purified NO-heme solutions (black and grey symbols; 100–200 µM NO-heme) by means of the standard addition method by using spiked nitrite standards (white symbols; 0–200 µM nitrite). Shown is the mean and standard deviation (SD) of three independent UV–Vis measurements

In order to subsequently remove nitrite and DHAA residues and thus produce purified NO-heme, 10 ml of the above-mentioned solution were dialyzed at 4 °C protected from light for 24 h against distilled and ultra-pure water using Slide-A-Lyzer^®^ dialysis cassettes (MWCO: 3500; Thermo Scientific, Dreieich, Germany). Purified NO-heme was then obtained as follows: in a first step, the solution in the dialysis cassette was removed and centrifuged at 2500 rcf for 10 min, leaving only precipitated NO-heme inside the cassette. In a second step, 8 ml pure acetone were injected into the empty dialysis cassette and subsequently removed once the NO-heme precipitate was (almost) completely dissolved. Thereafter, both solutions were combined, divided into 1.5 ml aliquots and dried for 90 min at 60 °C using a centrifugal vacuum concentrator (Concentrator 5301; Eppendorf, Hamburg, Germany). Lastly, all resulting pellets were dissolved and pooled in a total volume of 600 µl 80% acetone/H_2_O, flushed with nitrogen and stored at − 80 °C protected from light until further use. Immediately before the respective in vitro assays mentioned below were performed, NO-heme was quantified a second time and diluted to the desired concentration using 80% acetone/H_2_O. The cellular treatment with NO-heme was always performed in a darkened room with the lamp of the laminar flow cabinet as the only source of light.

To obtain a 4 mM hematin solution, 13.04 mg porcine hemin were dissolved in 1 ml 20 mM NaOH and diluted in 4 ml acetone. Thus, hematin contained the same acetone concentration as the NO-heme solutions, i.e. 80%.

### Photometric detection and quantification of heme species

All initial non-nitrosylated hemin as well as the NO-heme solutions were diluted in 80% acetone and their content was analyzed by UV–Vis spectroscopy in the range of 350–750 nm using a microplate reader (Infinite M200; Tecan, Crailsheim, Germany). The concentration of NO-heme was determined by applying the Beer–Lambert law and the published extinction coefficient of NO-heme, which is 11.3 mM^−1^ × cm^−1^ at 540 nm (Hornsey 1956).

### Fourier-transform infrared (FTIR) spectroscopy of heme species, reagents and reaction by-products

In order to further identify the hemin and NO-heme molecules present in the above-mentioned solutions, nitrogen-dried samples were analyzed in the range of 4000–400 cm^−1^ using an FTIR spectrometer (IRAffinity-1S; Shimadzu, Duisburg, Germany). Reference spectra of reagents and reaction by-products present in the NO-heme solution (e.g. DHAA, sodium nitrite and hemozoin [obtained from InvivoGen, Toulouse, France]) were recorded using the undissolved (i.e. dry) compounds.

### Stability analysis of NO-heme under different storage conditions

For the analysis of stability, NO-heme was stored in 15 ml centrifuge tubes (Greiner Bio-One, Frickenhausen, Germany) under different light and atmospheric conditions. Briefly, 80 ml of the NO-heme solution were first synthesized, purified and concentrated as described above. The final solution was further diluted in 80% acetone/H_2_O to a final volume of 12.5 ml and allocated to four centrifuge tubes containing 3 ml each. Two of the NO-heme samples were stored in the dark at room temperature, flushed with nitrogen and additionally sealed with thermoplastic film (Parafilm^®^ M, Sigma-Aldrich). The other samples were incubated at room temperature and exposed to sunlight and air. The NO-heme content of each tube was analyzed by UV–Vis and FTIR spectroscopy after 0, 2, 4 and 24 h of incubation.

### Nitrite content in NO-heme solutions

The residual nitrite concentration of the NO-heme solution was measured photometrically and calculated using the standard addition method, i.e. by adding increasing amounts of sodium nitrite to identical volumes and concentrations of the same NO-heme sample. This method enables the generation of a nitrite standard curve while avoiding matrix effects caused by compounds absorbing light at the same wavelength as the reaction reagents used to quantify nitrite (e.g. NO-heme itself). The colorimetric quantification of nitrite was performed as reported beforehand, albeit with slight modifications (Mohamed et al. 2008). NO-heme was diluted in ultra-pure water and mixed with 11.61 mM sulfanilamide (Sigma-Aldrich) and 2.51 mM *N*-(1-naphtyl)-ethylenediamine dihydrochloride (NED, Sigma-Aldrich) in 96-well plates (TPP, Trasadingen, Switzerland). Both reagents were dissolved in 0.5 M HCl, with solutions always being freshly prepared when needed. After the addition of NED, samples in the 96-well plates were incubated for 15 min at room temperature, followed by the measurement of the absorption at 540 nm. The absorption of the blank sample was determined by adding 0.5 M HCl instead of sulfanilamide and NED.

To estimate the nitrite content in NO-heme solutions after dialysis and vacuum concentration, nitrite standards (0, 100 and 200 µM concentrated in ultrapure water) as well as solutions of 100 and 200 µM NO-heme were spiked with nitrite and analyzed as described above. Thereafter, the nitrite standards were plotted in a diagram and used as indicators for nitrite detection in the NO-heme samples.

### SDS-PAGE and Western blotting

The cellular uptake of NO-heme and hematin was qualitatively investigated by using the expression of the intracellular heme-degrading protein heme oxygenase 1 (HO-1) as surrogate marker. Its expression was analyzed via SDS-PAGE and Western blotting in NO-heme-treated Caco-2 cells. Beforehand, the cytotoxicity of the heme species as well as solvents was evaluated using a commercial kit measuring cell viability based on the WST-1 reagent obtained from Roche Diagnostics (Mannheim, Germany) and performed according to the manufacturer's instructions. Briefly, 5 × 10^4^ cells/well were seeded in 96-well plates (TPP) and incubated for 24 h. Then, the cells were treated with the test substances in triplicate for a further 24 h before they were washed with phosphate buffered saline (PBS) and incubated for 1.5 h with 100 µl/well medium supplemented with 10% (v/v) WST-1 reagent. Finally, absorption was measured at 450 nm (with a reference wavelength at 650 nm). For HO-1 expression analysis, Caco-2 cells (3 × 10^5^/3.5 cm plate) were treated for 24 h as mentioned above, with 2% acetone/H_2_O and 2% acetone/NaOH serving as solvent controls. Subsequently, the supernatant and the PBS-washed cellular fraction were collected, pelleted with trypsinized cells and resuspended in 1× Lämmli loading buffer pre-heated to 95 °C. Proteins were then separated by SDS-PAGE and transferred onto a nitrocellulose membrane (Perkin Elmer, Rodgau, Germany) using a wet-blot chamber (BioRad, Munich, Germany) as previously described (Seiwert et al. 2017). After blocking the membrane in 5% non-fat dry milk (Carl Roth) in Tris-buffered saline/0.1% Tween-20 (TBS-T), incubation with primary antibodies occurred overnight at 4 °C. The membrane was then washed thrice in TBS-T followed by an incubation with secondary antibodies for 1 h at room temperature. After final washings steps, proteins were detected with enhanced chemoluminescence using an Azure 300 CL imaging system (Azure biosystems, Dublin, USA). Anti-heat shock protein 90 (Hsp90) *α/β* (mouse monoclonal; no. sc-13119; Santa Cruz Biotechnology, Heidelberg, Germany) and anti-heme oxygenase-1 (rabbit polyclonal; no. GTX101147, GeneTex, Irvine, California, USA) were used as primary antibodies. Horseradish peroxidase-conjugated secondary antibodies were purchased from Santa Cruz (anti-mouse) and Cell Signaling Technologies (anti-rabbit; Frankfurt am Main, Germany).

### Single cell electrophoresis assay (comet assay)

DNA-damaging effects of NO-heme were determined in Caco-2 and BALB/c 3T3 cells using the single cell electrophoresis assay (comet assay). The detection of genotoxicity in BALB/c 3T3 cells was thereby performed to enable a direct comparison of these results with effects documented in the BALB/c 3T3 cell transformation assay (see below). The comet assay was performed as previously reported (Briviba et al. 2016), with the following modifications: 2.15 × 10^4^ cells/well were seeded in 6-well plates (Corning, Kennebunk, USA) and cultivated for five days. At day six after seeding, the cells were treated for 1 h with the different test substances diluted in Hank’s Balanced Salt Solution (HBSS; Lonza). Hydrogen peroxide and iron sulfate (both from Sigma-Aldrich) served as positive controls. After treatment, the cells were prepared as previously published (Briviba et al. 2016) and incubated in a specific lysis buffer (100 mM Na_2_EDTA, 1% Triton X-100, 2.5 mM NaCl, 1% lauroyl sarcosine sodium salt, 10 mM tris(hydroxymethyl)aminomethane [TRIS] and 10% dimethyl sulfoxide; all purchased from Merck, Darmstadt, Germany) for 1 h at 4 °C. Then, microscope slides were incubated in electrophoresis buffer (1 mM Na_2_EDTA, 300 mM NaOH; Merck) for 20 min at room temperature, followed by an electrophoresis run at 25 V and 300 mA for 40 min. Thereafter, the slides were washed three times with TRIS buffer (0.4 M; pH 7.5) and stained using 85 µl of a 4,6-diamidino-2-phenylindole solution (5 µg ml^−1^; Merck). For quantification of the results, 100 cells per slide were analyzed using a DM 400 B fluorescence microscope (Leica, Wetzlar, Germany) connected to the Comet Assay II Image Analysis System (Perceptive Instruments, Halstedt, UK).

### Bacterial reverse mutation assay (Ames test)

The Ames test was performed according to the pre-incubation method described by Mortelmans and Zeiger (2000), albeit with slight modifications. Briefly, the lyophilized *Salmonella typhimurium* strains TA100 (lot number 5220D, Trinova, Gießen, Germany) and TA1535 (lot number 5209D, Trinova) were cultured overnight in 50 ml nutrient broth no. 2 (Thermo Scientific) at 37 °C. Subsequently, the bacterial cultures were shaken for two hours at 125 rpm and 37 °C until the suspension reached an optical density of 1–1.3 at 600 nm. Then, 100 µl of the suspension were mixed with 500 µl of sodium phosphate buffer (final concentrations: 12 mM NaH_2_PO_4_, 88 mM Na_2_HPO_4_ [pH 7.4]; all components purchased from Carl Roth) and 50 µl of the test compound. Sodium azide (Sigma-Aldrich) was used as positive control and dissolved in ultrapure water. The bacteria were mixed and incubated with the test compounds for 30 min at 37 °C prior to being plated and further incubated for 48 h at 37 °C. Finally, the number of grown revertant colonies was counted.

### In vitro mammalian cell gene mutation test (HPRT assay)

The investigation of potential cytotoxic effects induced by NO-heme in CHO-K1 cells was performed using a commercial cell proliferation kit based on the MTT reagent obtained from Roche Diagnostics and performed according to the manufacturer's instructions. Briefly, 3.5 × 10^4^ cells/well were seeded in 96-well plates (Greiner Diagnostic, Balingen, Germany). Approximately 24 h later, the cells were treated with different NO-heme and acetone concentrations for another 24 h. Subsequently, cells were incubated with the MTT solution for 4 h, before 100 µl DMSO (Carl Roth) were added and absorbance was measured at a wavelength of 570 nm using a microplate reader (EL600; BioTek Instruments, Winooski, USA). The mitochondrial metabolic activity of NO-heme-treated cells was thereby compared to the vehicle control. The HPRT assay was performed according to OECD test guideline no. 476 (OECD 2016) without exogenous metabolic activation. Briefly, 5 × 10^5^ cells per sample were seeded in a 75 cm^2^ cell culture flask (Greiner Bio-One) containing 15 ml of McCoy’s 5A medium. After 24 h, the cells were treated with the different test substances for 4 h, whereby ethyl methanesulfonate (EMS; Sigma-Aldrich) served as the mutation-inducing positive control. Then, the cells were washed with buffered saline, replenished in fresh medium and incubated for another 48 h. In order to evaluate the expression of the mutant phenotype, cells were sub-cultured every 48 h for 9 days. On day nine post treatment, 2 × 10^5^ cells were transferred in triplicate to new flasks, each containing 6-thioguanine (10 µg ml^−1^; Sigma-Aldrich). After another period of 9 days, colonies resistant to 6-thioguanine were fixed with methanol (Carl Roth), stained with methylene blue (Carl Roth) and counted. For the final evaluation of the test, the absolute number of mutations per one million CHO-K1 cells was calculated.

### BALB/c 3T3 cell transformation assay (CTA)

The appropriate concentrations of NO-heme to be used in the cell transformation assay were determined using a slightly modified colony forming efficiency (CFE) method according to Sasaki et al. (2012a). Briefly, 200 BALB/c 3T3 cells were seeded in 60 mm dishes (TPP) filled with 4 ml MEM medium. After 24 h, the cells were treated with NO-heme or the control substances for three days. 3-Methylcholanthrene (MCA, Sigma-Aldrich) dissolved in DMSO was used as positive control. On day 4, the medium was removed and 4 ml fresh culture medium were added. On day 9, the cells were washed with saline, fixed with 4 ml ice-cold methanol (Carl Roth) for 10 min and then stained with Giemsa solution (Carl Roth) for 30 min. Finally, the stained dishes were washed with water and air-dried before cell colonies with a diameter of ≥ 2 mm were counted.

The CTA was performed as previously described (Sasaki et al. 2012a) and began with the seeding of 2 × 10^4^ BALB/c 3T3 cells in 100 mm dishes (TPP) filled with 10 ml MEM medium. On day 1, the cells were incubated with the test substances for three days, followed by a medium change on day 4. From now on, the cells were incubated in DMEM/HAM’s F-12 medium supplemented with 2% (v/v) FCS, 100 µg ml^−1^ streptomycin, 100 IU ml^−1^ penicillin (all components were obtained from Biochrom) and 2 µg ml^−1^ bovine pancreas insulin solution (Sigma-Aldrich). On days 7, 10, 14, 17, 21 and 24, the medium was changed and the cells were finally fixed on day 31 using ice-cold methanol. Lastly, the plates were stained with Giemsa solution and the number of type III foci formed was counted using a stereo microscope (SZX2-ILLT; Olympus, Hamburg, Germany). Focus characterization and assessment were performed in accordance with the photo catalogue published by Sasaki et al. (2012b).

### Statistical analysis

All results were statistically analyzed using Prism (version 8.4.1; GraphPad, La Jolla, USA). Prior to the significance analysis (*α* = 0.05), the data were examined with respect to normality distribution by using the Shapiro–Wilk test. Detailed information on the number of replicates and the used statistic tests are mentioned in the legends of the corresponding Tables and Figures.

## Results

### Analytical characterization of heme species

NO-heme showed absorbance maxima at 480, 540 and 566 nm, whereas the non-nitrosylated hemin solution showed maxima at 510, 542 and 640 nm in the UV–Vis spectra (Fig. 1a). As depicted in Fig. 1b, the IR spectra of NO-heme exhibited bands at 2918.30, 2850.79, 1701.22, 1662.64, 1629.85, 1411.89, 1274.95, 1219.01, 950.91, 840.96, 704.02 and 476.42 cm^−1^. Although the spectrum of hemin was similar, with signals at 2916.37, 1697.36, 1662.64, 1408.04, 1278.81, 1209.37, 937.40, 844.82, 704.02 and 478.35 cm^−1^, NO-heme showed a maximum IR absorbance at 1662.64 cm^−1^, whereas hemin solutions featured an intensity maximum at 1697.36 cm^−1^. The IR spectra of hemin, acetone, sodium nitrite, ascorbic acid and DHAA were recorded in parallel and served as references to exclude interfering signals in the NO-heme spectra. As depicted in Fig. 1c, nitrite showed no bands in the range of 1500–1900 cm^−1^, while DHAA showed a maximum IR intensity at 1774.51 cm^−1^. In the case of ascorbic acid, bands at 1753.29 and 1654.92 cm^−1^ were documented. Similar to the IR spectra of hemin, acetone showed a maximum intensity at 1703.14 cm^−1^.

The efficiency of the NO-heme purification process was verified by quantifying the residual content of nitrite in the synthesized solutions. Non-purified 1 mM NO-heme solutions contained 102.7 ± 10.7 mM nitrite (mean ± SD; *n* = 6) or 10.27 mM nitrite when diluted in a 100 µM NO-heme solution. In comparison, a 100 µM NO-heme solution subjected to dialysis and vacuum concentration contained amounts of nitrite below the LOQ (100 µM) of the nitrite quantification assay (Fig. 1d). This degree of purification corresponds to a higher than 100-fold dilution of nitrite in NO-heme solutions produced by the above-mentioned modified protocol.

### Stability of NO-heme solutions

**Fig. 2:**
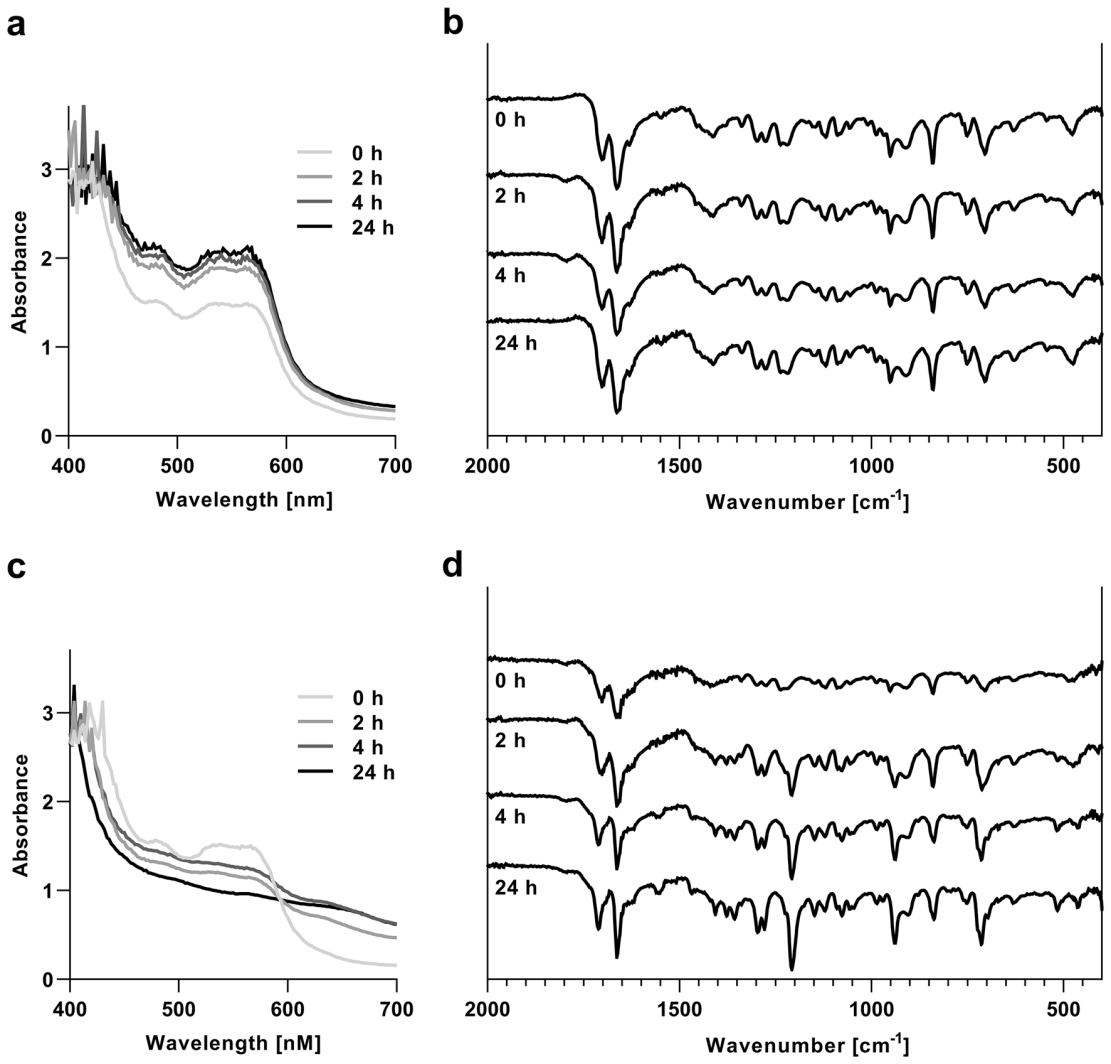
Stability of NO-heme under different lighting and atmospheric conditions after 0, 2, 4 and 24 h of incubation. **a** UV–Vis and **b** IR spectra of NO-heme stored in the dark under a nitrogen atmosphere. **c** UV–Vis and **d** IR spectra of NO-heme exposed to sunlight under normal atmospheric conditions (i.e. air)

**Fig. 3:**
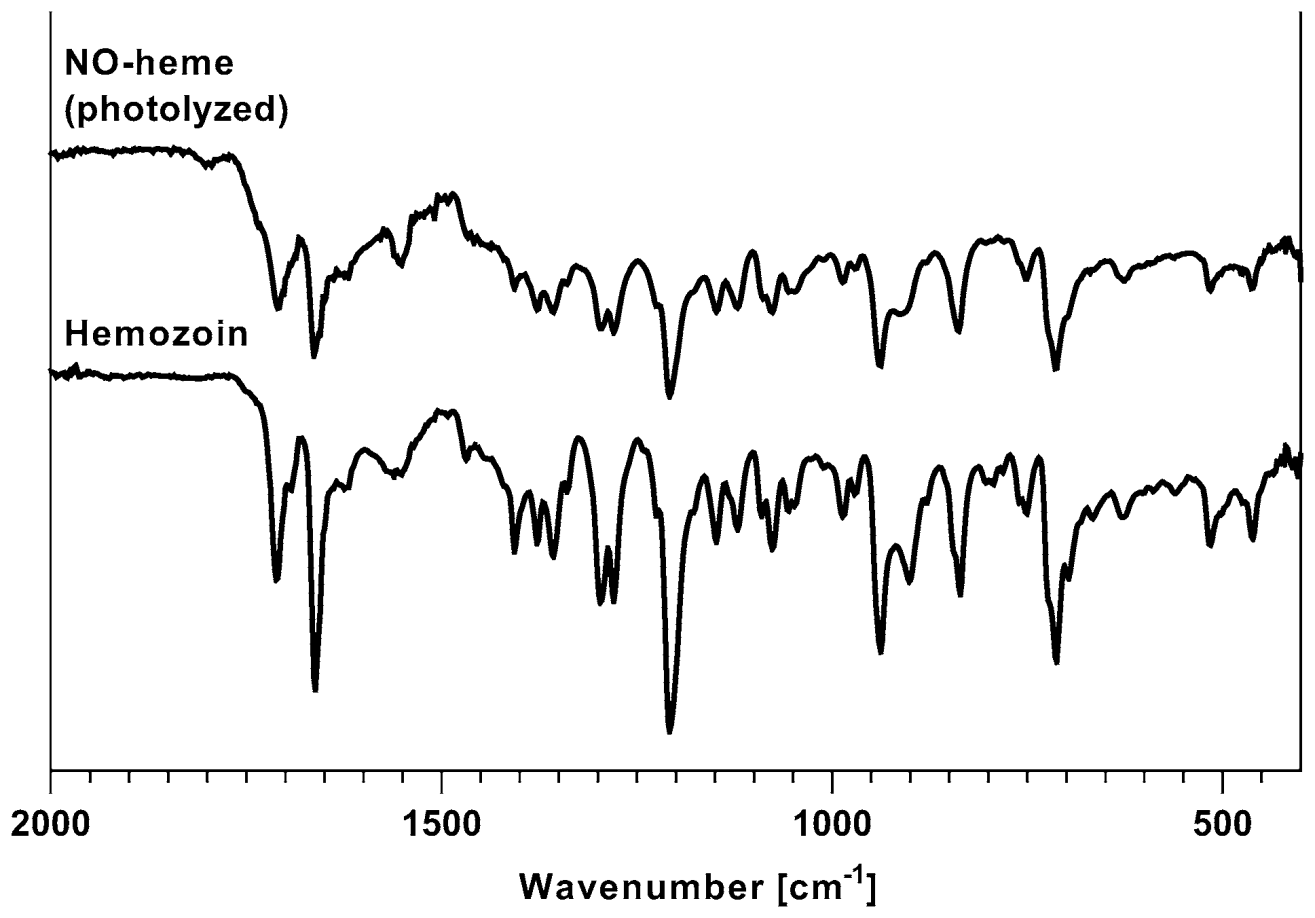
Analysis of photolyzed NO-heme. The IR spectrum of NO-heme after exposure to sunlight and air for 24 h in comparison to the IR spectrum of hemozoin

The stability of NO-heme solutions, which were protected from light and flushed with nitrogen, was not affected over an incubation period of up to 24 h according to the UV–Vis and IR spectra depicted in Fig. 2a and b. Storage at -80 °C additionally helped to maintain stability for at least eight weeks, as depicted in Supplementary Fig. 2. Exposure of NO-heme solutions to UV light and oxygen reduced the respective absorbance signals in the UV–Vis spectrum within two hours of exposure (Fig. 2c), while additional IR bands emerged at 1207.44 and 1554.63 cm^−1^ (Fig. 2d). In addition to these newly appeared bands, the color of the exposed NO-heme solution changed from red to brown and a precipitate was formed, which consisted of brown crystalline structures. IR spectra of these crystals were identical to the IR spectrum of the heme dimer hemozoin (Fig. 3).

### Effects of the heme species in different in vitro assays

**Fig. 4:**
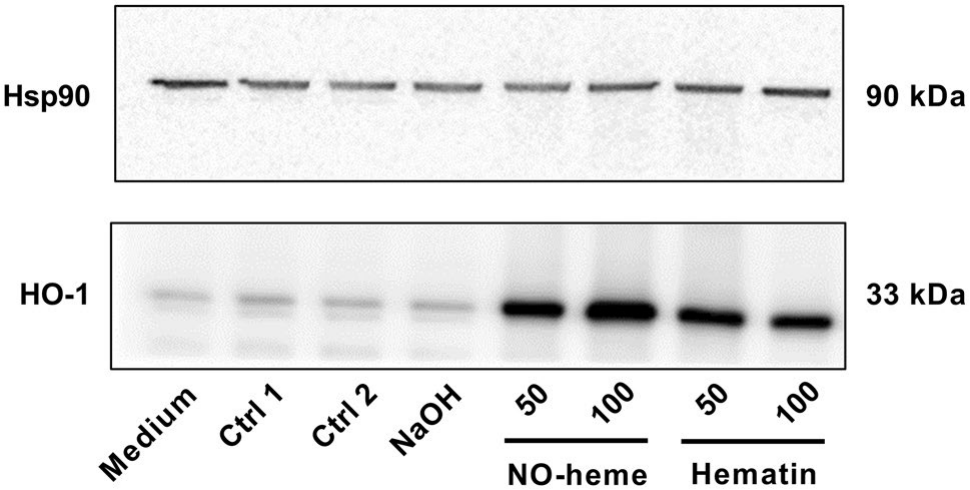
Expression of HO-1 in Caco-2 cells exposed to NO-heme (50–100 µM) and hematin (50–100 µM) for 24 h. The solvent control of hematin (Ctrl 1) and NO-heme (Ctrl 2) were diluted with cell culture medium in the same way as the heme species, with the final test concentration being 2% acetone/NaOH in case of Ctrl 1 and 2% acetone/H_2_O in case of Ctrl 2. In addition, NaOH mixed with ultra-pure water instead of acetone was tested. Hsp90 served as loading control

Prior to the analysis of potential toxic effects of hematin and NO-heme, their cellular uptake was determined using the heme-degrading protein heme oxygenase 1 (HO-1) as marker. The experiments showed nearly the same results for both test solutions, i.e. when compared to control samples, treatment with 50 and 100 µM hematin and NO-heme, respectively, resulted in an increased expression of HO-1 during the 24-h incubation period (Fig. 4). Similar to the HO-1 expression analysis, a dose-dependent increase of cytotoxicity was seen with comparable effects in Caco-2 cells treated with both heme species (50 µM NO-heme and hematin reduced the relative cell viability to 43% and 45% of the control, respectively; Supplementary Fig. 3a).

**Fig. 5:**
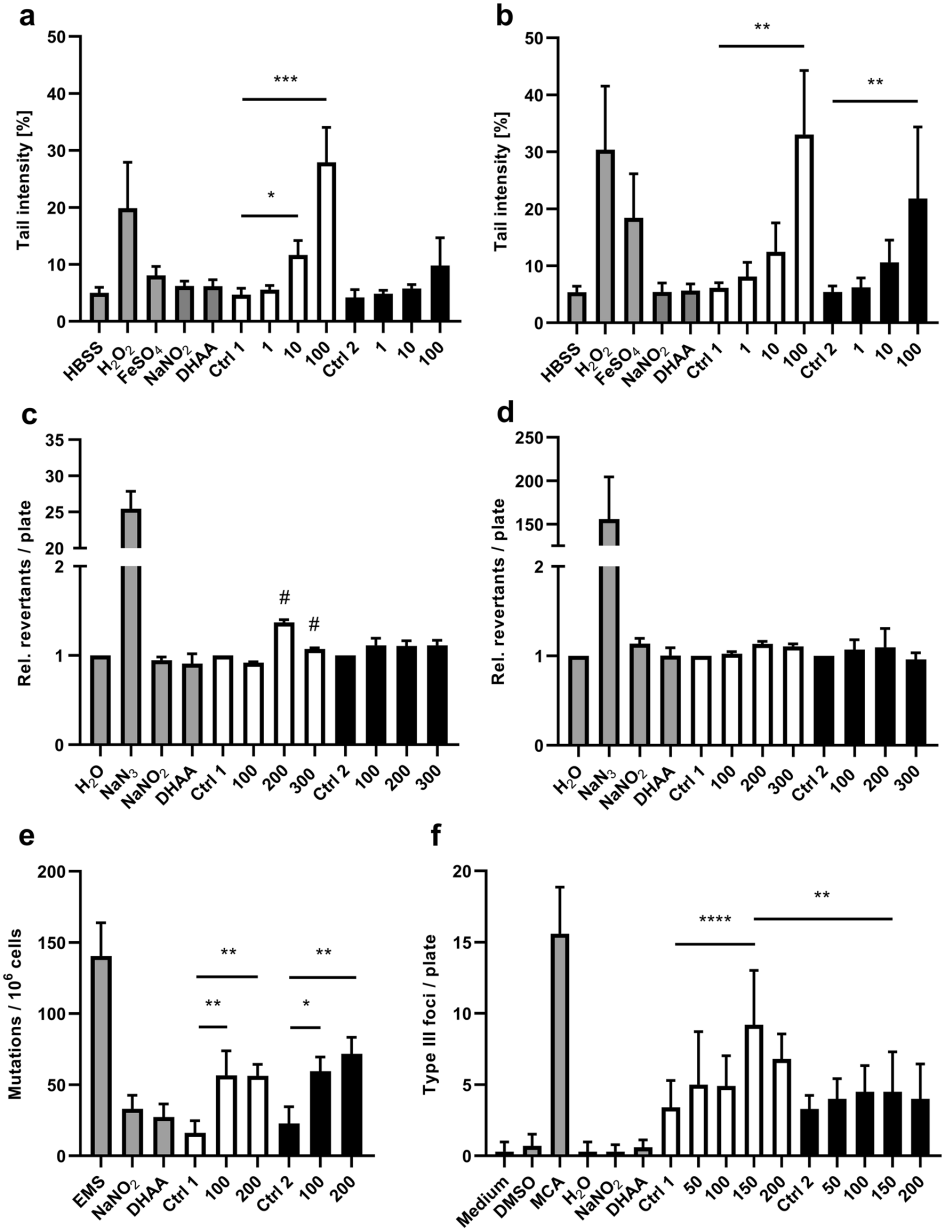
Toxicological characterization of hematin (white bars; 1–300 µM hematin dissolved in 80% acetone/NaOH [Ctrl 1]) and NO-heme (black bars; 1–300 µM NO-heme dissolved in 80% acetone/H_2_O [Ctrl 2]) using various in vitro assay systems. Shown is the mean and SD of at least three independent experiments, with the exception of the comet assay performed with Caco-2 cells (*n* = 4) and the BALB/c 3T3 cell transformation assay (*n* = 10). Statistical analysis was performed using the Kruskal–Wallis test followed by Dunn’s multiple comparison post-hoc analysis in the case of comet assay data as well as the Ames test performed with *S. typhimurium* strain TA1535. The Ames test performed with *S. typhimurium* TA100 as well as the HPRT and the BALB/c 3T3 cell transformations assays were analyzed by means of a one-way ANOVA followed by Tukey’s multiple comparison test. **a** Comet assay performed with BALB/c 3T3 cells. **b** Comet assay performed with Caco-2 cells. **c** Ames test performed with *S. typhimurium* strain TA100. **d** Ames test performed with *S. typhimurium* strain TA1535. **e** HPRT assay performed using CHO-K1-BH4 cells. **f** BALB/c 3T3 cell transformation assay. For all assays, the solvent controls Ctrl 1 and Ctrl 2 were adapted to the highest NO-heme or hematin concentration ranging from 2% in the comet assay to 4% in the HPRT as well as the BALB/c 3T3 cell transformation assays and 6.15% acetone/H_2_O or acetone/NaOH in the Ames tests. The concentrations of the control substances were as follows: 100 µM NaNO_2_, 61.54 µM DHAA, 100 µM H_2_O_2_, 100 µM FeSO_4_, 1.54 mM NaN_3_, 7.69% H_2_O in the Ames tests, 5 mM EMS, 0.1% DMSO, 14.91 µM MCA and 0.2% H_2_O in the case of BALB/c 3T3 cell transformation assay. *p < 0.05, **p < 0.01, ***p < 0.001, ****p < 0.0001. ^#^Occurrence of cytotoxic effects visually determined by the absence of a background cell monolayer

The genotoxic potential of NO-heme and hematin was analyzed in the comet assay using BALB/c 3T3 and Caco-2 cells. Cell viability was assessed using a live/dead staining after the 1-h treatment, a necessary step for cell counting and seeding on the microscope slides. By doing so, no cytotoxicity was detected for any test substance (data not shown). In the comet assay using BALB/c 3T3 cells, hematin led to a dose-dependent increase of the tail intensity with statistically significant effects in the concentration range of 10–100 µM (Fig. 5a), while the increase in tail intensity induced by NO-heme was generally lower than that induced by hematin and not statistically significant. In contrast, Caco-2 cells reacted more sensitively to the treatment, exhibiting genotoxic effects when exposed to increasing hematin and NO-heme concentrations: incubation of Caco-2 cells with 100 µM NO-heme thereby led to a tail intensity increase of 22%, while the same amount of hematin reached a mean tail intensity of 33% (Fig. 5b). For lower concentrations, a statistically significant DNA-damaging potential was not seen, although a dose-dependent tendency was noticed.

Except for the positive control NaN_3,_ no mutagenic effects were detected in the Ames test, although concentrations of up to 300 µM NO-heme and hematin were tested (Fig. 5c, d). Prior to the performance of the HPRT assay, the cytotoxicity of NO-heme and the solvent acetone/H_2_O was analyzed in CHO-K1 cells using the MTT assay. The treatment of CHO-K1 cells with NO-heme led to a dose-dependent decrease of the relative cell viability with values ranging from 87% viability for 50 µM NO-heme to 59% viability for 200 µM NO-heme, while increasing concentrations of acetone/H_2_O did not affect cellular viability (Supplementary Fig. 3b). The results of the HPRT assay are shown in Fig. 5e. 100 µM as well as 200 µM NO-heme and hematin induced significantly higher frequencies of mutations in CHO-K1 cells when compared to the solvent controls. The effects of both heme species were thereby similar, with a mutation rate of 56–71 mutations per 10^6^ cells.

In a last step, the potential of both heme species under investigation to induce a malignant transformation of BALB/c 3T3 cells was analyzed. A concentration of 150 µM hematin, but not NO-heme, induced a significant increase in the amount of type III foci when compared to the corresponding solvent control (Fig. 5f). Moreover, treatment of cells with 150 µM hematin induced a significantly higher number of type III foci when compared to 150 µM NO-heme. In spite of these rather high concentrations, BALB/c 3T3 cells showed no sign of cytotoxicity up to the maximum concentration of 200 µM used for both heme species. In contrast, both DHAA and sodium nitrite showed no cell-transforming potential, although cytotoxicity was seen starting at fairly high concentrations of 750 µM and 6 mM, respectively (Supplementary Fig. 3c–f).

## Discussion

To our knowledge, this is the first study describing a reliable method for the chemical synthesis of purified and stable NO-heme with a significantly reduced level of contaminants and by-products such as nitrite. The UV–Vis spectra of hemin and NO-heme measured herein were identical with the spectra measured by Hornsey (1956), Shahidi and Pegg (1991) and Soltanizadeh and Kadivar (2012), with specific absorbance maxima at 476–480, 535–540 and 563–566 nm. IR spectra of hemin and NO-heme showed similar bands from 3000 to 1700 cm^−1^ and 1600 to 400 cm^−1^. Therefore, it can be concluded that these bands are specific for the heme molecule. In contrast, the intensities at 1662.64 cm^−1^ differ considerably between the two heme species, suggesting a difference in the iron ligand most probably due to the binding of a nitric oxide moiety to the heme molecule. In the case of hemin, the band at 1662.64 cm^−1^ may confirm the existence of multiple carbon–carbon double bonds within the heme structure (Morin et al. 1989; Silva et al. 1999), while in the case of NO-heme, this (low-intensity) band could be overlapped by a band specific to the Fe–NO bond and whose intense stretching appeared at the same wavenumber. These results are supported by several earlier studies analyzing heme nitrosylation, in which similar IR signals in the range of 1678–1659 cm^−1^ were reported, and in which the IR band was specifically attributed to the Fe–NO bond (Killday et al. 1988; Jankiewicz et al. 1994; Pegg and Shahidi 1996). Moreover, in the present study, the interference of bands by solvents and by-products was excluded by measurement of reference spectra, whereas sodium nitrite and DHAA as well as acetone showed no concurrent bands with the signal at 1662.64 cm^−1^ specifically attributed to the Fe–NO bond in the frame of the present study. Furthermore, the NO-heme solution was measured in the dry state and showed no band at 1774.51 cm^−1^, rendering the concurrent detection and thus the presence of acetone and/or DHAA unlikely, even more when considering that the starting NO-heme solution was purified prior to measurement. Ascorbic acid showed a band at 1654.92 cm^−1^; therefore, one could speculate that the NO-heme band seen at 1662.64 cm^−1^ is due to contamination of the NO-heme solution with ascorbic acid. However, the second band at 1753.29 cm^−1^, which is specific for ascorbic acid (Bichara et al. 2015), was not seen, greatly diminishing the possibility of interfering bands resulting from ascorbic acid contamination of the NO-heme solution. Therefore, the presented IR spectrum is most likely specific for NO-heme, strongly suggesting that the method used for its synthesis and purification described herein is suitable for the reliable production of purified NO-heme.

As described by Hornsey (1957), Soltanizadeh and Kadivar (2012) and Haile et al. (2013), NO-heme is highly sensitive to UV light and oxygen; an exposure to these results in NO release from the molecule and leads to changes in the specific UV–Vis and IR spectra within 2 h. While the absorbance maxima of the UV–Vis spectra disappeared during photolysis, the IR spectra expanded with further bands appearing at 1554 and 1207 cm^−1^. In conjunction with the nitrosyl iron-related band at 1662.64 cm^−1^ as well as the emergence of brown crystalline structures, the appearance of these new bands support the assumption that the product of photolyzed NO-heme may be β-hematin or hemozoin. While the term “hemozoin” exclusively describes the heme species synthesized by parasites responsible for causing malaria (e.g. *Plasmodium falciparum*), synthetic hemozoin is termed β-hematin (Vanderesse et al. 2016). β-Hematin is a molecule that consists of two heme moieties joined by two ester linkages (chemical structure shown in Supplementary Fig. 1b) (Coronado et al. 2014; Vanderesse et al. 2016). While one propionate side chain of one heme molecule is connected to the iron atom of the other molecule, the second side chain can generate intermolecular hydrogen bonds with other β-hematin molecules (Egan 2002; Jaramillo et al. 2009). Thus, insoluble crystal structures are formed (Butykai et al. 2013; Coronado et al. 2014; Vanderesse et al. 2016), leading to the formation of the precipitate observed after NO-heme exposure to sunlight. In addition, β-hematin-specific IR bands in the range of 1664–1660 cm^−1^ as well as 1211–1207 cm^−1^ attributed to the ester linkages have been described in several previous studies (Slater et al. 1991; Egan et al. 1994; Basilico et al. 1997; Huy et al. 2007; Jaramillo et al. 2009; Coronado et al. 2014; Vanderesse et al. 2016). Therefore, in spite of the light-induced release of the NO moiety, the band at 1662.64 cm^−1^ is still detected due to ester bonds existing between the remaining heme molecules. Finally, the photolysis-induced formation of β-hematin was verified by comparison of sunlight-exposed NO-heme solutions with IR spectra of pure hemozoin, resulting in identical wavenumber bands for both substances. All in all, these results strongly suggest that β-hematin is formed after the light-induced release of NO from the NO-heme molecule, as reported by Ostera et al. (2011). Based on the analytical results presented herein, toxic effects induced by possible contaminants and by-products resulting from the nitrosylation reaction used to produce the NO-heme solution are highly unlikely. Hence, all the toxic effects described herein for the purified NO-heme solution can be attributed to the main molecule under investigation, i.e. NO-heme.

The cytotoxicity of NO-heme was analyzed prior to the in vitro examination of its genotoxic, mutagenic and cell-transforming potential. The analyses using BALB/c 3T3 cells showed either no cytotoxicity (for NO-heme and hematin) or a reduced cell viability being induced only by a more than tenfold higher concentration than that used in the comet assay and the CTA (in case of DHAA and sodium nitrite). In the CHO-K1 cells used for the HRPT test, NO-heme-induced cytotoxicity and reduced the relative cell viability to 59%. However, the OECD test guideline no. 476 (OECD 2016), describing the performance of the HPRT assay, allows for the use of test compound concentrations reducing cellular viability to 10–20% of control. Likewise, for the Western blot analysis on cellular uptake, Caco-2 cells were treated with hematin and NO-heme at concentrations that induced cytotoxicity after 24 h of incubation concurrently to the corresponding comet assays performed after 1 h of treatment with these compounds. Thus, the toxic effects seen may be interpreted as a consequence of DNA damage occurring at the early stages of the exposure of the cells to hematin and NO-heme, while the increased expression of HO-1 strongly indicates that both heme species were indeed taken up by the Caco-2 cells.

One may speculate that the DNA-damaging and mutagenic effects induced by both tested heme species is caused by increased lipid peroxidation. Generally, each lipid molecule found in the cell membrane consists of unsaturated fatty acids, which could react with reactive oxygen species (ROS) to form lipid hydroperoxides (Marnett 2000; Ayala et al. 2014). Besides these primary peroxidation products, reactive aldehydes like 4-hydroxynonenal (4-HNE) or malondialdehyde (MDA) can be formed as secondary products (Ayala et al. 2014). These can directly interact with DNA and form several DNA adducts (Stone et al. 1990a, 1990b; Guéraud et al. 2010). Further genotoxic effects induced by 4-HNE are extensive DNA fragmentation, sister-chromatid exchanges and chromosome aberrations (Brambilla et al. 1986; Esterbauer et al. 1990; Eckl et al. 1993). A sum of all these toxic effects may finally result in the malignant transformation of cells exposed to 4-HNE. The heme molecule and especially the iron atom can indeed trigger the formation of hydroxyl radicals through the so-called Fenton reaction (Ayala et al. 2014) and may thus activate pathways leading to the above-mentioned genotoxic effects, as indicated by the increased formation of ROS in cells treated with different heme species (Goldstein et al. 2003). Moreover, in in vivo studies using rodents, the consumption of heme-enriched diets led to increased levels of thiobarbituric acid-reactive substances, whose content correlates with the MDA concentration (Tsikas 2017) in the colon and feces of the test animals (Pierre et al. 2004, 2007, 2008; Toden et al. 2010; Ijssennagger et al. 2013; Bastide et al. 2015) as well as with a significantly higher occurrence of DNA strand breaks in isolated colonocytes (O'Callaghan et al. 2012). Genotoxic effects previously seen in cells treated with heme (Glei et al. 2002; Glei et al. 2006; O'Callaghan et al. 2012) are in line with the results seen in the comet assay and suggest that these DNA strand breaks may arise from DNA adducts caused by heme-induced MDA and/or 4-HNE formation. This mechanism could also be responsible for the mutagenicity detected in the HPRT assay performed in the present study. In the case of MDA, Cajelli et al. (1987) were able to show a mutagenic potential emanating from this compound in the HGPRT assay, which is a test system similar to the one employed herein, albeit using V79 Chinese hamster lung fibroblasts. Moreover, Gilsing et al. (2013) previously described mutagenic effects resulting from heme intake derived from red meat consumption in an epidemiological study performed in The Netherlands and linked heme intake with a higher incidence of G → A transition mutations in colorectal tumors. ROS formation induced by heme species resulting in the formation of DNA adducts and mutations could also be responsible for the malignant transformation seen in the CTA. The hematin-induced formation of type III foci infers that this molecule may be involved in processes increasing cell proliferation and suppressing the cell–cell contact inhibition normally inherent to BALB/c 3T3 cells (Sasaki et al. 2012b). Although the test compounds were able to induce genotoxic effects in test systems using mammalian cells, both NO-heme and hematin failed to induce any effects in the Ames test. An explanation for this finding could be either the absence of nitrosylable compounds in the treatment buffer needed for the formation of potentially mutagenic NOCs after NO release from NO-heme, or the absence of bacterial heme uptake mechanisms. The latter hypothesis is supported by data published by Arimoto et al. (1980), which showed that the original non-modified *S. typhimurium* strain is unable to take up heme, an ability most probably also lacking in the strains used in the present study (TA100 and TA1535).

It has been hypothesized that NO-heme induces stronger toxic effects when compared to non-nitrosylated heme, mostly due to the reactivity of the NO moiety. For example, during protein fermentation in the colon, NO might react with glycine, one of the most abundant amino acids in food (Harrison et al. 1997), to first form *N*-nitroso glycine and, in a next step, after a dehydration reaction, yield diazoacetate (Cupid et al. 2004; Gottschalg et al. 2007). Diazoacetate is highly reactive and may bind to DNA, resulting in the formation of DNA adducts such as *O*^6^-methylguanine or *O*^6^-carboxymethylguanine (Shuker and Margison 1997; Anderson et al. 1999; Cupid et al. 2004; Lewin et al. 2006; Gottschalg et al. 2007). However, as shown in the present study, the presence of an NO moiety in the heme molecule does not induce effects beyond those seen for heme alone, which questions our initial hypothesis and suggests that the observed effects are principally mediated by the heme moiety.

While heme and iron can act as pro-oxidants and may induce ROS formation (Sergent et al. 1997; Goldstein et al. 2003; Puri et al. 2012), nitric oxide can, interestingly, act as an antioxidant, which, upon reaction with an oxidizing agent, results in the generation of nitrite (Kanner 1994; Hogg and Kalyanaraman 1999; Sharpe et al. 2003). Moreover, the pro-oxidative effects of cysteine and hemoglobin can be easily modulated by nitrosylation processes emerging from the antioxidative compounds nitrosocysteine and nitrosohemoglobin (Kanner 1979; Kanner et al. 1992). Application of this concept to the experimental setup presented here may speculatively explain the results obtained, i.e. the fact that NO-heme does not induce stronger genotoxic effects than hematin. During the treatment of cells with NO-heme, denitrosylation may occur, thereby leading to the release of heme. This molecule may in turn increase ROS formation, while the released nitric oxide may counteract the deleterious effects of ROS, as has been shown for nitrosomyoglobin by Kanner et al. (1980). The genotoxic effects induced by NO-heme may thereby occur independently of the NO moiety, as the heme molecule may still induce DNA damage via heme or β-hematin-initiated lipid peroxidation processes as described above (Schwarzer et al. 1992; Carney et al. 2006; Schrimpe and Wright 2009; Ayala et al. 2014).

While nitrosylation processes promote the formation of NOCs, which may possess antioxidant properties (Kanner 1979; Kanner et al. 1980, 1992), NO itself is known to induce DNA strand breaks and mutations in mammalian cells (Isomura et al. 1984; Nguyen et al. 1992; Tamir et al. 1996). In spite of positive results regarding genotoxicity and mutagenicity, both Nguyen et al. (1992) and Tamir et al. (1996) hypothesized that not NO, but rather more complex nitrogen oxides (NOx), formed in the presence of oxygen, could be responsible for these effects. Furthermore, Szaleczky et al. (2000) questioned the toxicity of NO and suggested that genotoxic and mutagenic effects seen in cells upon treatment with NO may be attributed to the formation of NOCs or reactive ions such as the peroxynitrite anion (ONOO^−^), which can be formed by the reaction of NO with a superoxide radical (O_2_^−^; Liu and Hotchkiss 1995; Lundberg et al. 2008; Kundu and Surh 2012; Habermeyer et al. 2015). In the presence of oxygen, NO may also be converted to nitrogen dioxide (NO_2_), a reactive intermediate that subsequently may lead to the emergence of nitrite and nitrate (Liu and Hotchkiss 1995; Lundberg et al. 2008; Habermeyer et al. 2015). However, as summarized by Szaleczky et al. 2000 and reviewed by Habermeyer et al. 2015, the mechanisms underlying the toxic and or carcinogenic effects of NO, nitrite, nitrate as well as their NO-related products are largely unknown, due to the instability of nitrous and oxygen radicals and their so far unexplored complex reactions leading to the formation of several DNA-reactive NOCs. Speculatively, the above-mentioned chemical reactions may have also occurred in the frame of the in vitro assays used in the present study. However, although a reaction of NO with oxygen species after release of the NO moiety from the heme molecule cannot be ultimately excluded, it is unlikely that the formation of complex nitrogen oxides underlies the effects described herein. For instance, at least a tenfold higher concentration of peroxynitrite (1 mM) is needed to induce DNA strand breaks when compared to NO-heme (100 µM; Tamir et al. 1996).

However, it has to be mentioned that nitrite, as a reaction product of NO with ROS, may be formed and trigger mutagenic effects similar to those caused by the intact NO-heme molecule. For example, it has previously been shown that nitrite is mutagenic in the HPRT assay and leads to the malignant transformation of BALB/c 3T3 cells at concentrations ranging from 1–10 mM (Kodama et al. 1976; Tsuda and Hasegawa 1990; Stevanovic et al. 2000), although it did not induce DNA single strand breaks in murine cell lines (Kodama et al. 1976; Görsdorf et al. 1990). Under the assumption that all existing NO moieties of NO-heme reacted with ROS to form nitrite, a maximum concentration of 1–300 µM nitrite could be reached in the different incubations of the present study. Therefore, mutagenic and cell-transforming effects such as those previously described are unlikely. In this context, it is important to mention that each assay included a 100 µM nitrite control, which showed no genotoxic, mutagenic or cell-transforming effects.

In summary, 10–150 µM heme, independently of the NO moiety, induced a dose-dependent formation of DNA strand breaks as well as gene mutations and was able to cause malignant cell transformation. An important factor in the overall assessment of these data is the comparability of the tested heme concentrations with dietary concentrations prevailing in the gut after red meat consumption. The amount of heme found in the gut after red meat consumption depends on the following factors: the colon volume as well as the amount and type of meat ingested, with the latter showing high variations in heme content depending on the animal species under study. In the case of beef, the heme content varies from 12 to 105 µg g^−1^ (Mistura and Colli 2009; Pretorius et al. 2016), while poultry meat contains 1.6–6 µg g^−1^ (Vanden Bussche et al. 2014; Pretorius et al. 2016), resulting in an average heme content of about 59 µg g^−1^ for beef and 4 µg g^−1^ for poultry. In order to calculate heme uptake, in which case additional influencing factors such as the bacterial uptake of heme are not considered (Yilmaz and Li 2018), an average colon volume of 561 ml is assumed (Pritchard et al. 2014). Therefore, the consumption of 100–500 g meat would theoretically result in an intestinal heme concentration of 1–6 µM for poultry and 17–85 µM for beef, which, in the case of the latter, is well in the range of the concentrations used in the present study (10–200 µM).

## Conclusion

A reliable method for the synthesis of pure and stable NO-heme was established, which enabled a preliminary toxicological characterization of this cured-meat pigment. The initial hypothesis that the NO-heme molecule shows a higher pro-carcinogenic potential than non-nitrosylated heme could not be confirmed. The present results rather suggest that the heme molecule alone may promote tumor formation, especially when considering that heme concentrations inducing effects in vitro are in the lower range of concentrations occurring in the colon in vivo after the consumption of a typical amount of red meat. Heme thus appears to be one of the most important factors in the frame of red meat-associated CRC, although nutrition-related CRC should be considered a multifactorial event rather than being only attributed to a single causal element (reviewed by Demeyer et al. 2016).

## Electronic supplementary material

Below is the link to the electronic supplementary material.

Supplementary file1 (PDF 760 kb)
