# Supplementary material for: Synthesis and in vitro characterization of the genotoxic, mutagenic and cell-transforming potential of nitrosylated heme
Source: Arch Toxicol. 2020 Jul 15;94(11):3911–27. doi: 10.1007/s00204-020-02846-8 (PMC7603461; doi:10.1007/s00204-020-02846-8)
Supplement: Supplementary file 1 — Supplementary file1 (PDF 760 kb) [file 204_2020_2846_MOESM1_ESM.pdf]

Synthesis and *in vitro* characterization of the genotoxic, mutagenic and cell-transforming potential of nitrosylated heme

Tina Kostka[1, 2, *], Jörg Fohrer[3], Claudia Guigas[4], Karlis Briviba[4], Nina Seiwert[5], Jörg Fahrer[5], Pablo Steinberg[1, 4], Michael T. Empl[1]

[1] *Institute for Food Toxicology, University of Veterinary Medicine Hannover, Hannover, Germany*

[2] *Current address: Institute of Food Science and Human Nutrition, Leibniz University Hannover, Hannover, Germany*

[3] *Institute of Organic Chemistry, Leibniz University Hannover, Hannover, Germany*

[4] *Max Rubner-Institut, Federal Research Institute of Nutrition and Food, Karlsruhe, Germany*

[5] *Division of Food Chemistry and Toxicology, Department of Chemistry, Technical University of Kaiserslautern, Kaiserslautern, Germany*

[*] Corresponding author

Email address: kostka@lw.uni-hannover.de

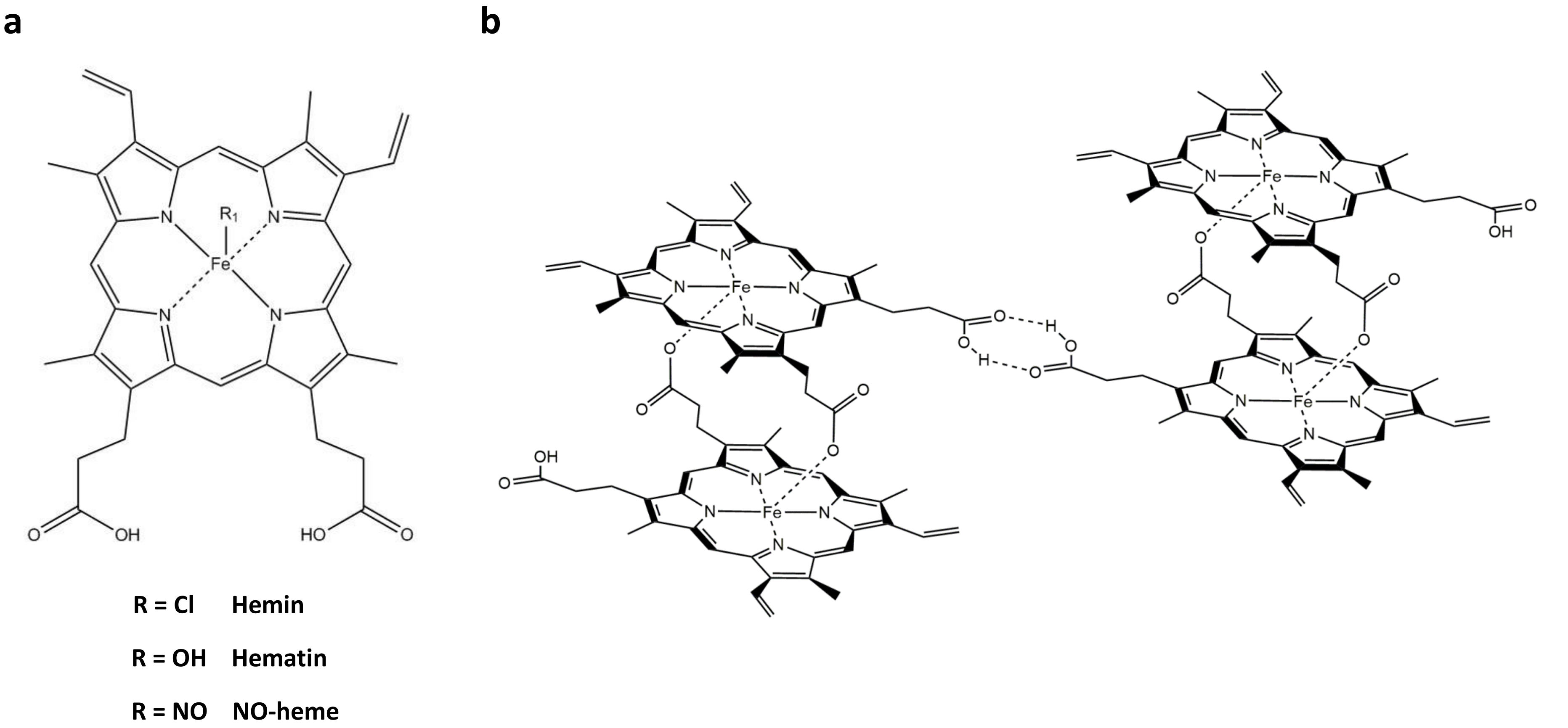

Supplementary Fig. 1 Chemical structure of different heme species. **(a)** Heme monomer with different iron ligands specific for the substances hemin, hematin or NO-heme. **(b)** Dimeric structure of hemozoin/β-hematin

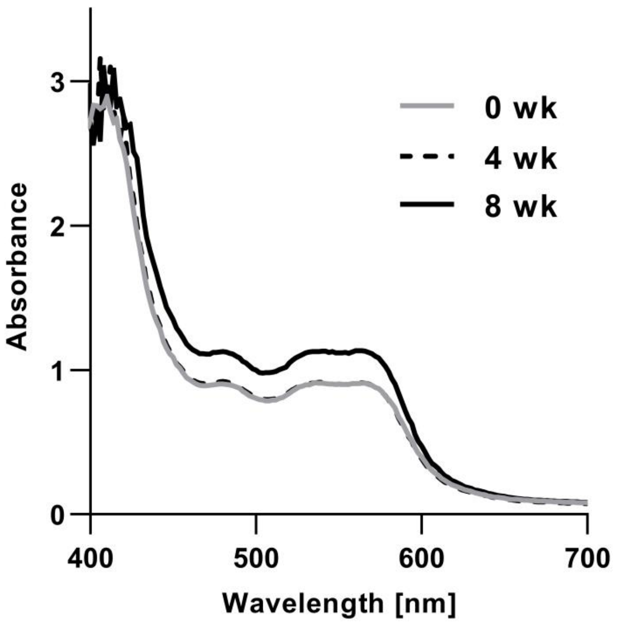

Supplementary Fig. 2 UV-Vis spectra of NO-heme stored in the dark under a nitrogen atmosphere at -80 °C over 0, 4 and 8 weeks (wk)

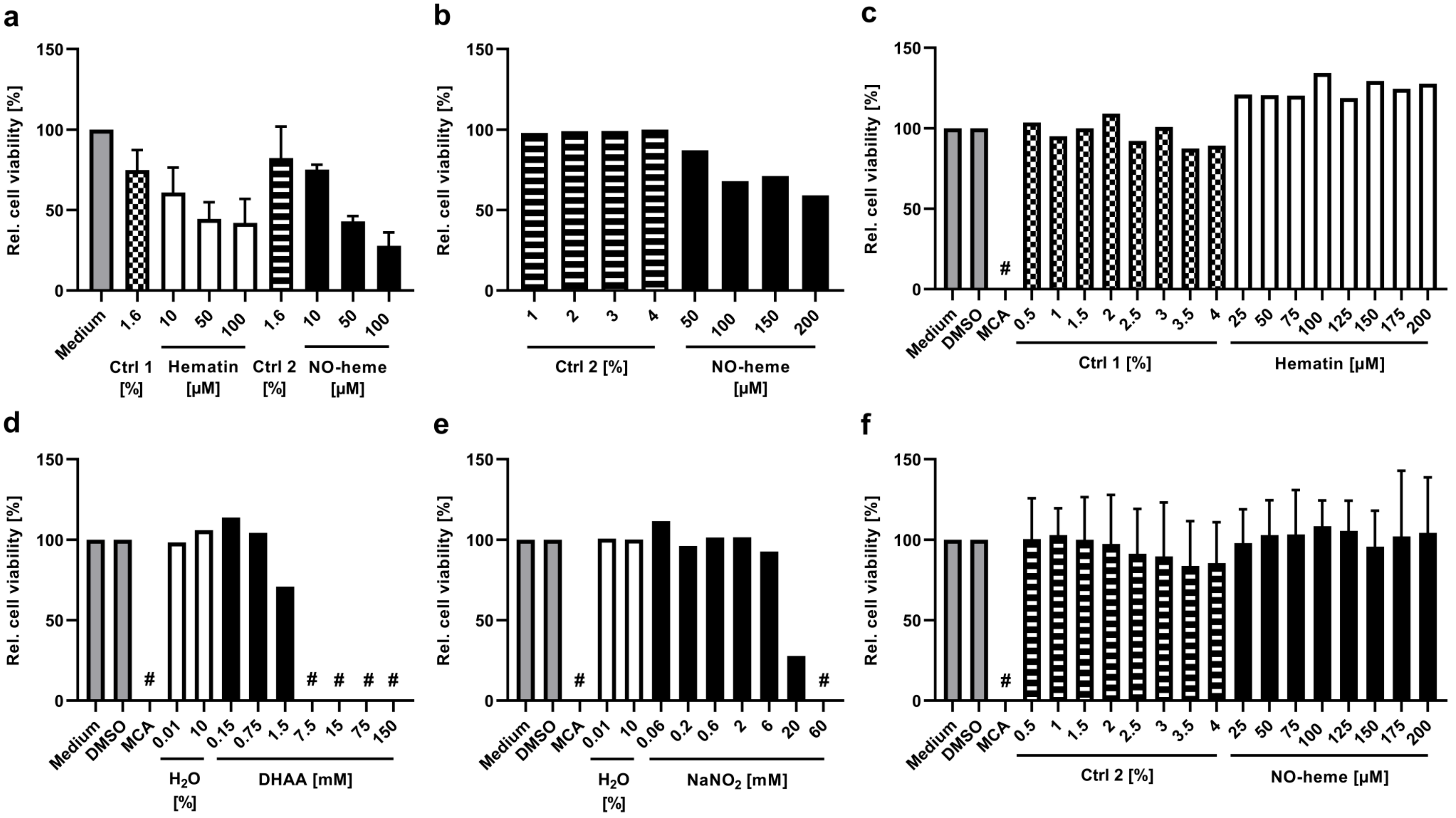

Supplementary Fig. 3 Cytotoxicity of NO-heme and hematin as well as the solvents acetone/NaOH (Ctrl 1), acetone/H_2O (Ctrl 2) and the control substances DHAA and $NaNO_2$. (**a**) WST-1 assay using Caco-2 cells. Shown is the mean and SD of 3 (NO-heme) or 4 (hematin) independent experiments, whereby all samples were compared to the culture medium control. (**b**) MTT assay performed using CHO-K1 cells. Shown is the mean of 2 independent experiments, whereby all results were compared to the highest solvent control (4 % Ctrl 2). (**c-f**) CFE assay performed using BALB/c 3T3 cells. Shown is the mean of 2 independent experiments (NO-heme: mean and SD of 3 independent experiments), whereby all samples were compared to the medium control except for MCA (4 µg/ml), which was compared to its solvent control containing 0.1 % DMSO. #: The relative cell viability ranged from 0 to 1 % and is therefore not visible in the figures
